# Influence of deep marginal elevation on the marginal adaptation and fracture resistance of zirconia endocrowns constructed on endodontically treated premolar teeth: in vitro study

**DOI:** 10.1007/s44445-025-00044-9

**Published:** 2025-08-05

**Authors:** Sara Tamimi, Huda I. Mostafa, Menna S. Ali, Hayam M. Tawfik

**Affiliations:** 1 Department of Crowns and Bridges, Faculty of Dental Medicine for Girls, Al-Azhar University, Cairo, Egypt; 2 Department of Endodontics, Faculty of Dental Medicine for Girls, Al-Azhar University, Cairo, Egypt; 3 Department of Operative Dentistry, Faculty of Dental Medicine for Girls, Al-Azhar University, Cairo, Egypt

**Keywords:** Endodontically treated teeth, Endocrowns, Ceramics, Deep marginal elevation, Fracture resistance

## Abstract

**Introduction:**

One of the most conservative approaches to restoring teeth that have undergone endodontic treatment is the use of endocrowns. This study evaluated the marginal adaptation and fracture resistance of endodontically treated premolars restored with endocrowns fabricated from translucent multi-layered zirconia ceramic following deep marginal elevation.

**Methods:**

Eighteen freshly extracted intact human maxillary premolars were endodontically treated, and standard endocrown preparations were performed. Specimens were divided into two groups (n = 9 each) based on the location of the proximal margin. Group I: Butt-joint endocrown design with a cavity 2 mm in width and 1 mm above the cemento-enamel junction (CEJ) prepared at the middle of the mesial surface (without deep marginal elevation). Group II: Butt-joint endocrown design with a cavity 2 mm in width and 2 mm below the CEJ prepared at the middle of the mesial surface. Deep marginal elevation (DME) was performed using composite resin. All endocrowns were fabricated from translucent multi-layered zirconia ceramic. Endocrowns were luted using dual-cure adhesive cement. Thermocycling was performed after incubating the samples in distilled water at 37°C for 24 h. Marginal adaptation was examined using a stereomicroscope (× 40) at three specific locations along the mesial border. Fracture resistance was assessed using a universal testing machine.

**Results:**

No statistically significant difference was detected in marginal adaptation or fracture resistance between the two groups (p = 0.121 and 0.589, respectively). Catastrophic failure (fracture below the CEJ) was more frequently observed in both groups.

**Conclusion:**

DME has no significant effect on either marginal adaptation or fracture resistance of premolars restored with multi-layered translucent zirconia endocrowns. DME slightly improved the failure mode.

## Introduction

Restoring endodontically treated premolars remains a challenge in restorative and prosthetic dentistry due to the increased biomechanical risk of failure. Teeth may fracture because of trauma, deep dental caries, endodontic and restorative procedures, or a reduction in water content and structural integrity (Zhang et al. 2021). Maximum tooth fragility is observed in teeth with deep proximal defects that have undergone endodontic treatment. Full-coverage restorations have been recommended for endodontically treated premolars to reduce the risk of fracture (Ali and Moukarab 2020).

Posts were advised in cases where restorations lacked sufficient retentive surfaces due to the loss of coronal tooth structure. However, this treatment option remains controversial, as creating a post space weakens the already fragile tooth structure and may result in root fracture, especially in teeth with thin roots (Bresser et al. 2019).

The endocrown procedure is considered a less invasive alternative to the post-and-core approach. Utilizing both the axial walls of the pulp chamber and adhesive luting types of cement, the monoblock porcelain is retained macro-mechanically and micro-mechanically, respectively. It is regarded as a less invasive method that provides crown retention with minimal preparation and is considered a more straightforward procedure compared to other methods (Mahjoubi et al. 2023; Arafa and Ziada 2023).

Hassouneh et al. (2020) concluded that CAD/CAM resin composite endocrowns could be an optimal choice for restoring endodontically treated premolars. Thomas et al. (2020) found that the success rates of endocrowns in molars and premolars were nearly identical, suggesting premolars as suitable candidates for endocrowns. Chen et al. (2022) reported in a 46-month study that the success rate of endocrowns was comparable to that of traditional crowns for posterior endodontically treated teeth. Abd El Rhman et al. (2022) concluded that zirconia endocrowns exhibited higher fracture resistance than PEEK and E.max endocrowns. However, PEEK endocrowns showed a more favorable failure mode, thereby preserving the tooth structure. Dawood et al. (2021) evaluated the effect of various bonding methods and bonding substrates on the marginal integrity of lower molars restored with monolithic zirconia restorations following thermocycling. They found that the bonding substrate, whether dentin, enamel, or composite, can influence the marginal quality of zirconia restorations.

When proximal defects are located subgingivally, it becomes challenging to apply rubber dams, perform adhesive cementation, control marginal microleakage, achieve the dry conditions required for cementation, and take accurate impressions. A less invasive technique was introduced to elevate the cavity margins supragingivally by applying a layer of composite that is subsequently covered by an indirect ceramic restoration (Robaian et al. 2023). This method, known as proximal box elevation (PBE), utilizes a suitable resin composite to elevate the intracervical margin to the supragingival level, thereby facilitating rubber dam isolation after the cavity margins have been repositioned (Ghajghouj and Taşar-Faruk 2019; Theodora et al. 2022).

Ali et al. (2020) reported that the fracture resistance and marginal adaptation of Vita Enamic and IPS e.max CAD were improved after deep marginal elevation. Zhang et al. (2021) concluded that proximal box elevation of maxillary premolars that were endodontically treated and restored with ceramic endocrowns improved fracture resistance; however, no corresponding reduction in microleakage was observed. Robaian et al. (2023) concluded that when a greater amount of tooth structure was involved, fracture resistance decreased even with monolithic zirconia crowns. However, the deep marginal elevation technique up to 2 mm below the cemento-enamel junction (CEJ) did not adversely affect fracture resistance.

Polymerization of composite resin is commonly associated with substantial shrinkage, which may lead to microleakage, secondary caries, marginal staining at the tooth-restoration interface, and cuspal deflection, resulting in adhesive failure (Theodora et al. 2022).  Several studies have shown that when endodontically treated premolars are restored indirectly, marginal adaptation and fracture resistance may be influenced by the use of a resin composite layer for proximal box elevation. This research aimed to assess the fracture resistance of endocrowns made of translucent multi-layered zirconia ceramic constructed on endodontically treated premolar teeth following deep margin elevation.

## Materials and methods

### Sample size calculation

To compare two groups of premolar teeth that had undergone endodontic treatment and were restored with endocrowns made of translucent multi-layered zirconia ceramic and to assess the effect of using composite to elevate the deep margin on marginal adaptation and fracture resistance, an ANOVA test was employed (Jaykaran and Tamoghna 2013). The mean fracture load (N) ranged from 1385.2 (± 186.7), 1154.1 (± 311.2), and 1083.6 (± 397.1) to 1446.9 (± 195.4), as reported in a prior study by Zhang et al. (2021). According to the guidelines provided by Zhang et al. (2021) and the sample size calculation using the G*Power statistical power analysis program (version 3.1.9.4), a total of 18 samples, with 9 in each group, would be sufficient to detect a large effect size (f = 0.64). This calculation assumes a two-sided hypothesis test with a statistical power of 80% (1 – β = 0.8) and a significance level of 5% (α = 0.05).

### Teeth collection

A total of eighteen premolars with double roots, extracted as part of orthodontic treatment plans, were selected for inclusion in this study. This was appraised and approved by the Research Ethical Committee (REC) under code (REC-PD-24–08). Patients provided written consent prior to extraction.

Teeth exhibiting enamel abnormalities, fractures, fissures, or any other defects were excluded from the investigation. Following disinfection in 5% sodium hypochlorite and removal of soft tissue and dental calculus, the teeth were preserved in a 0.1% thymol solution ("Caelo, Hilden, Germany") and then maintained in distilled water at 37 °C for no more than one month prior to use in the study.

A preoperative radiograph was taken of the selected teeth to confirm the absence of anatomical variations, ensuring that each root contained a single root canal. A digital caliper was used to measure all selected teeth at the CEJ to record the bucco-lingual and mesio-distal dimensions. Root length was measured. No significant variation was found between the groups. Specimens that did not match the specified measurements were replaced.

### Endodontic treatment

For standardization, all endodontic treatments were performed by the same operator following a consistent protocol and using the same instruments. A round carbide bur ("BR-31, Mani, Japan") was used to prepare the access cavity. The Protaper system ("Dentsply, Maillefer, Switzerland") was used to prepare the root canals, and an endo motor equipped with controlled torque ("X-smart, Dentsply, Sirona, Switzerland") was used. Working length was determined using radiographs. All canals were prepared up to file F3 of the Protaper system. Sodium hypochlorite 2.5% (5 mL) was used for irrigation between each file, followed by 17% EDTA (5 mL) as a final irrigant for smear layer removal, and then 5 mL of saline to reduce the chelating effect of EDTA. Protaper paper points were used to dry the canals.

A bioceramic-based sealer ("Total Fill, USA") was used in this study. Obturation was performed using the single-cone technique with a gutta-percha point matching the corresponding size of the Protaper system ("Dentsply, Maillefer, Switzerland"). A hot plugger was used to trim the excess gutta-percha 2 mm below the orifice. The canal orifice was then sealed with 2 mm thickness flowable composite ("Ivoclar, Vivadent, Germany") to enhance the bonding of the endocrowns. Postoperative radiographs were taken to evaluate the length, density, and taper of the root canal filling. Specimens were stored in distilled water at 37 °C for 24 h to ensure complete setting of the obturation material.

### Specimens grouping

Specimens were randomly categorized into two groups by assigning each specimen a designated number from 1 to 18, inscribed on paper. Each number was then randomly allocated to one of the two groups using a manual stratification method. Using a centralizing device, the specimens were fixed in epoxy resin blocks with self-curing, solvent-free, transparent epoxy (Kemapoxy 150, modulus 12 GPa), which resembles the structural integrity of human bone, in an upright position at a level 4 mm below the CEJ.

The specimens were divided into two groups, each comprising nine specimens, based on the position of the proximal margins. Group I consisted of butt-joint endocrowns without marginal elevation, with the mesial marginal cavity positioned in the center of the mesial surface, 2 mm in width, and located 1 mm above the CEJ. Group II involved butt-joint endocrown designs with deep marginal elevation, where the mesial marginal cavity was centered on the mesial surface, 2 mm in width, and located 2 mm below the CEJ. The mesial proximal cavity was meticulously prepared at the center of the mesial surface by delineating a line along its midpoint and subsequently extending the cavity by 1 mm on either side of this line to achieve a final cavity width of 2 mm at the gingival margin. The buccal and lingual walls of the proximal box were slightly flared to achieve passive path of insertion and removal to have the final width occlusally to be 4 mm.

### Endocrown and proximal box preparation

All specimens received a standardized endocrown preparation, with a 90° butt margin design used for the occlusal preparation. Cavity preparation was performed with minimal dentin removal to eliminate all pulp chamber undercuts and to adjust the internal axial walls.

A 1 mm elevation of the cervical floor of the proximal box above the CEJ level was performed. As part of the matrix-in-a-matrix approach, a modified circumferential matrix was used. A sectional matrix was placed within the modified circumferential matrix, with Teflon tape positioned between the two matrices to adjust the subgingival matrix. A 37% phosphoric acid solution ("BISCO, Etch-37, USA, 1–847-534–6000, Lot number 1–800-247–3368") was applied to etch the proximal box for 15 s, then rinsed for an additional 15 s and gently air-dried. The adhesive ("BISCO, All-Bond Universal, USA") was applied for 20 s, gently air-dried to vaporize the solvent, and then light-cured for 10 s. A thin layer of flowable composite (Tetric EvoFlow) was used to cover the cervical floor of the proximal box, followed by regular composite (Tetric N-Ceram), which was then packed over it and cured for 15 s resulting in a total elevation height of 3 mm. The thickness of composite was verified using a digital caliper. Restoration surfaces were finished and polished. All procedures were performed by two operators who received standardized training to ensure consistency in technique, particularly in the execution of deep marginal elevation (DME).

### Endocrowns fabrication and cementation

An extraoral scanner was used to scan all specimens for designing the endocrowns using CAD software. All endocrowns were fabricated from translucent monolithic multi-layered Katana zirconia blocks. The zirconia endocrowns were milled ("Kuraray Noritake Dental Inc., Japan"), sintered, stained, and glazed. Each endocrown was placed on its corresponding tooth and examined for complete seating and marginal adaptation. The fitting surfaces of all endocrowns were thoroughly cleaned using an ultrasonic cleaner for three minutes, rinsed under running water, and allowed to dry. Sandblasting of the fitting surfaces was performed using 50 μm aluminum oxide particles, followed by priming with Z-Prime Plus.

Cementation was carried out according to the manufacturer's instructions. Enamel borders of the prepared surfaces were etched with 37% phosphoric acid for 15 s, rinsed for 15 s, and air dried. All-Bond Universal Adhesive was used to prime the prepared surfaces. Two coats of adhesive were applied with 20 s of rubbing, followed by light air drying for 5 s to evaporate the solvent and light curing for 10 s. Specimens were cemented using dual-cure self-adhesive resin cement ("BISCO, Duo-Link Universal, USA"). Mixing and application were performed following the manufacturer's guidelines, and the endocrowns were affixed to each appropriately prepared specimen. A constant load of 2 kg was applied for 5 min using a designated loading apparatus. Initial light curing was performed for 2 s, after which excess luting cement was removed with a scaler. Final light curing was then performed for 20 s. To facilitate bond maturation, specimens were stored in distilled water at 37 °C for 24 h.

### Thermocycling procedure

Following the guidelines set out by the International Organization for Standardization (ISO/TS 11405), all specimens were subjected to 500 cycles of thermocycling in a water bath with a temperature range of 5 °C to 55°C. Each cycle lasted 30 s, with a 5-s transfer interval between each pair of baths.

### Evaluation of marginal adaptation

**Fig. 1:**
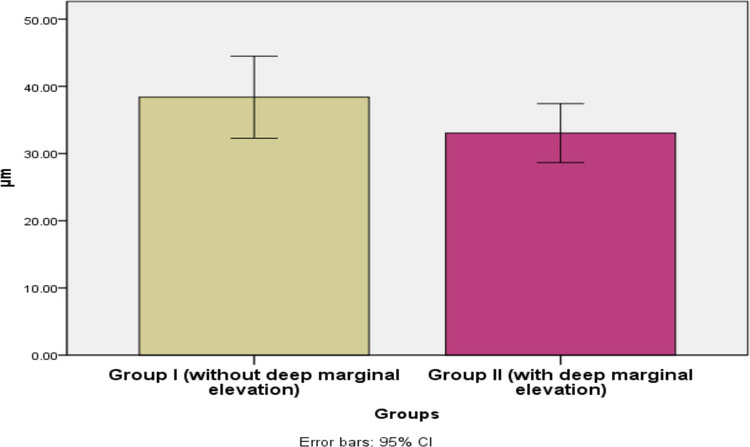
A bar chart illustrating the mean marginal gap (µm) in endocrowns bonded to enamel without DME versus with DME

Four stereomicrophotographs were taken of each specimen using a stereomicroscope (Wild 400, Switzerland) at 40 × magnification to evaluate marginal adaptation. The images were transferred to a computer system using image analysis software (Image Pro-Plus V6). One operator who was blinded to the respective groups was assigned to measure the gaps. The cervical margin of the mesial surface was measured at three evenly spaced, predetermined points along the cervical margin at the endocrown–composite interface: one at the exact center of the margin, and two additional points located 0.5 mm mesially and distally from the central point (Fig. 1), followed by calculation of the mean gap for each specimen in micrometers (μm) for statistical analysis.

### Testing fracture resistance

Fracture resistance was tested using a universal testing machine ("Lloyd LRX, Lloyd Instruments, Fareham, Hants, UK"). Each specimen was mounted in the device's lower compartment. A compressive load of 0.5 mm/min was applied along the long axis of the specimen using a stainless steel rod with a 6 mm round-ended tip, mounted on the upper arm of the testing machine. A sheet of tin foil was placed between the loading tip and the specimen to distribute stress evenly and minimize localized force spikes. The load was applied in a manner that ensured contact with both the lingual and facial cusp inclinations, targeting a point located in the center of the occlusal surface (central fossa). Vertical loading was continued until fracture occurred, and the fracture resistance was recorded in Newtons.

### Failure mode analysis

The stereomicroscope was set at 30 × magnification ("MA 100 Nikon stereomicroscope, Japan") with Omnimet image analysis software to evaluate and record the failure mode. Photographs of the fractured surfaces were captured for additional analysis. The evaluator assessing failure modes was blinded to group allocation to minimize assessment bias.

Failure modes observed were classified as follows:

I. Endocrown fracture without displacement: cohesive failure.
II. Endocrown debonding without fracture: adhesive failure.
III. Endocrown fracture with displacement: cohesive-adhesive failure.
IV. Endocrown and tooth fracture above the CEJ.
V. Endocrown and/or tooth fracture below the CEJ requiring tooth extraction.

### Statistical analysis

Statistical analysis and data management were performed using SPSS version 20. Summary statistics were calculated using mean, standard deviation, median, range, and confidence intervals. To assess the normality of the data distribution, the Shapiro–Wilk and Kolmogorov–Smirnov tests were applied. The marginal gap and fracture resistance values were compared between groups using an independent t-test, assuming normal distribution. The chi-square test was used to compare the qualitative data between groups, which were reported as counts and percentages, and to determine the failure mode. All p-values were two-sided, and results were considered statistically significant at p ≤ 0.05.

## Results

### Marginal adaptation

A higher mean value was recorded in Group I (endocrowns bonded to enamel without DME) (38.39 ± 7.95 µm) compared to Group II (endocrowns bonded to composite with DME) (33.04 ± 5.71 µm). Despite this variation, the results did not reach statistical significance (p = 0.121) (Table 1, Fig. 1).

**Table 1:** Marginal gap (µm) in endocrowns bonded to enamel without DME versus with DME (independent t-test)

Groups	Mean	Std. Dev	Std. Error	Median	95% Confidence Interval for Mean		Min	Max	t	p
					Lower Bound	Upper Bound				
**Group I**	**38.39**	**7.95**	**2.65**	**38.39**	**32.27**	**44.50**	**27.69**	**47.69**	**1.64**	**0.121** **ns**
**Group II**	**33.04**	**5.71**	**1.90**	**33.04**	**28.65**	**37.43**	**25.15**	**41.29**		

Significance level p ≤ 0.05, ns: non-significant

### Fracture resistance

**Fig. 2:**
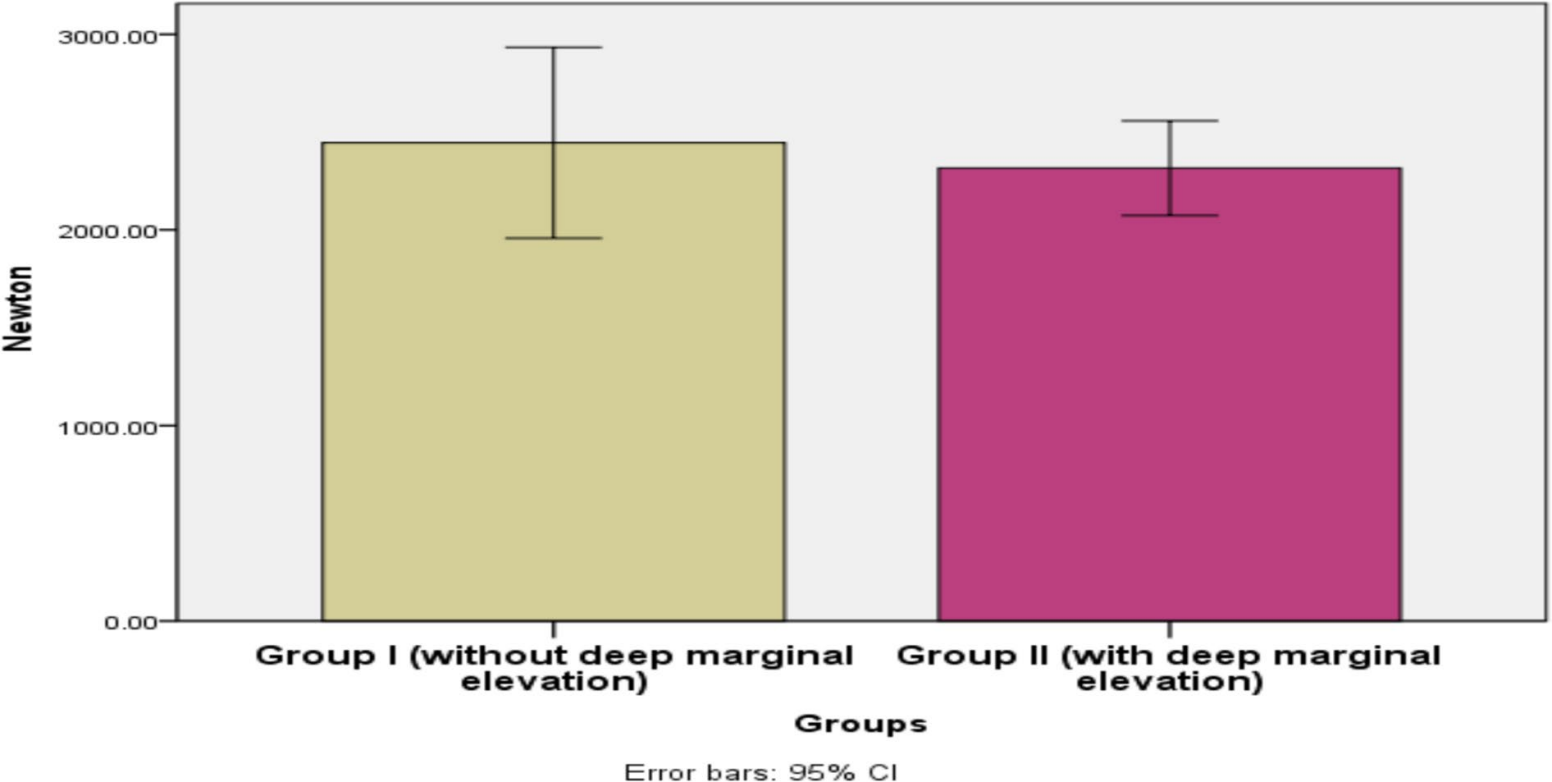
A bar chart illustrating the mean maximum load of fracture resistance (Newton) in endocrowns bonded to enamel without DME and with DME

A higher mean value was recorded in Group I (endocrowns bonded to enamel without DME) (2445.65 ± 635.23 N) compared to Group II (endocrowns bonded to composite with DME) (2315.39 ± 314.61 N). Despite this variation, the results did not reach statistical significance (p = 0.589) (Table 2, Fig. 2).

**Table 2:** Maximum load of fracture resistance (Newton) in endocrowns bonded to enamel without versus with DME (independent t-test)

Groups	Mean	Std. Dev	Std. Error	Median	95% Confidence Interval for Mean		Min	Max	t	p
					Lower Bound	Upper Bound				
**Group I**	**2445.65**	**635.23**	**211.74**	**2445.65**	**1957.36**	**2933.93**	**1698.01**	**3433.08**	**0.55**	**0.589 ns**
**Group II**	**2315.39**	**314.61**	**104.87**	**2315.39**	**2073.56**	**2557.22**	**1899.73**	**2747.60**		

Significance level p ≤ 0.05; ns: non-significant

### Failure mode

**Fig. 3:**
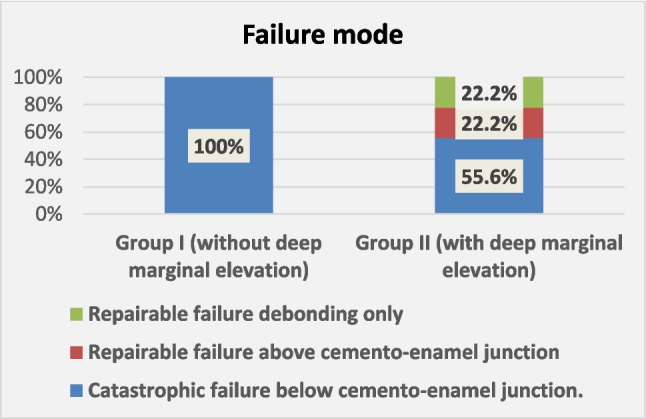
A bar chart illustrating the frequency of failure modes in both groups

**Fig. 4:**
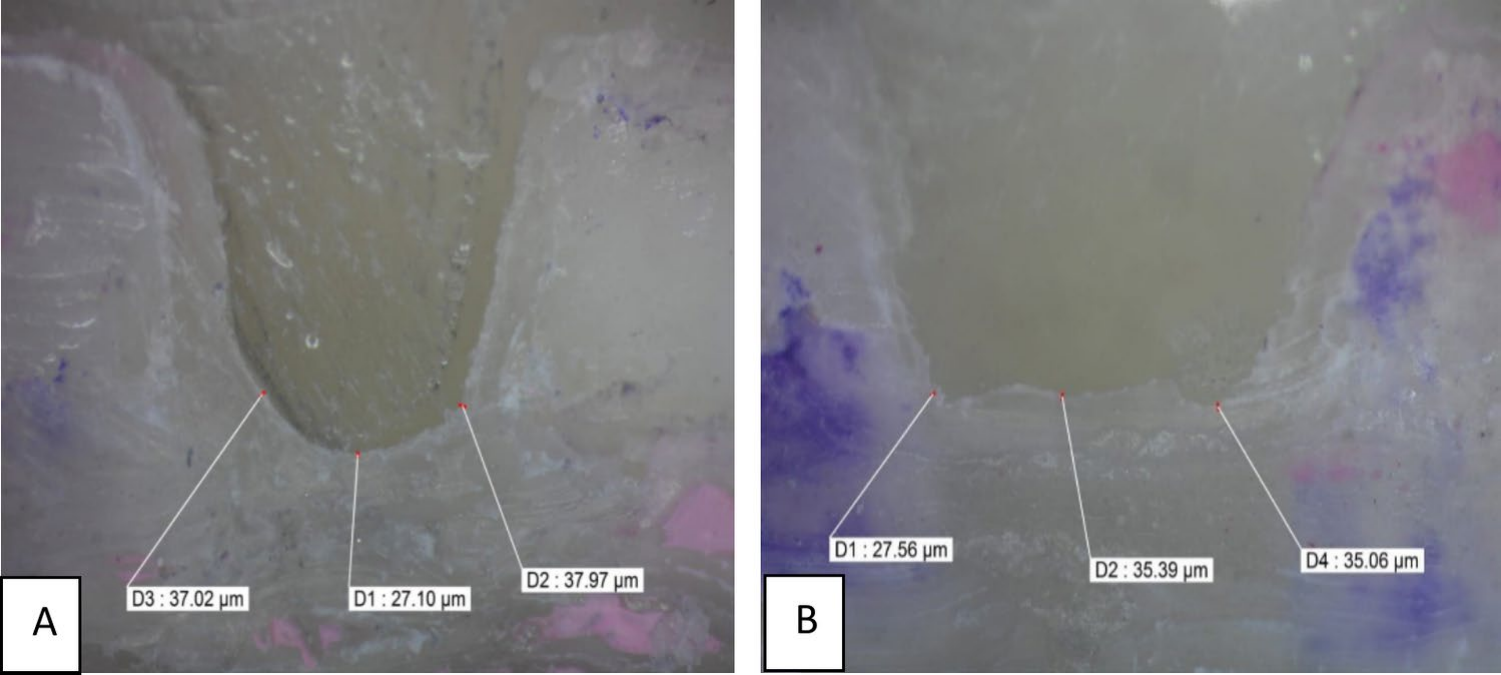
Stereo-microphotograph at a magnification of 40 × for both groups: (**A**) for Group I and (**B**) for Group II

**Fig. 5:**
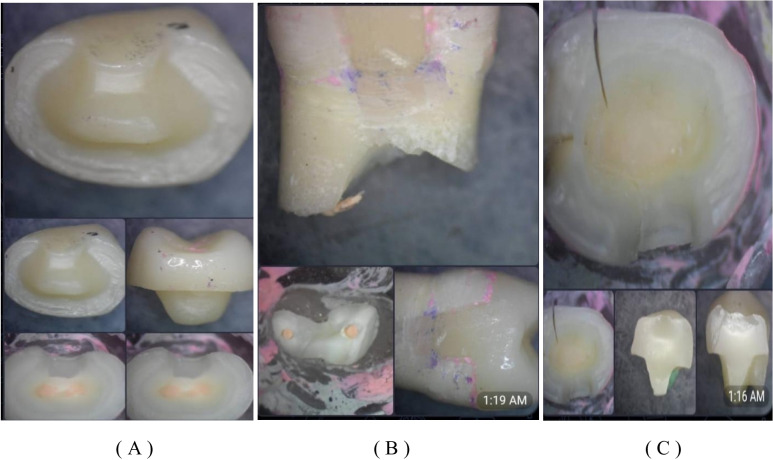
Failure modes observed under the stereomicroscope. (**A**) adhesive failure, (**B**) catastrophic fracture below the CEJ, and (**C**) repairable fracture above the CEJ

Group I (endocrowns bonded to enamel without DME) showed 100% catastrophic failure below the CEJ. In contrast, Group II (endocrowns bonded to composite with DME) exhibited 55.6% catastrophic failure (fracture below the CEJ), 22.2% repairable failure (fracture above the CEJ), and 22.2% repairable failure (debonding only). The results did not reach statistical significance (p = 0.55) (Figs. 3, 4 and 5).

## Discussion

High-quality coronal restorations that preserve good marginal integrity are essential for the long-term success of endodontically treated teeth. These restorations restore function and maintain the residual tooth structure. Because endodontic restorations often require the removal of additional tooth structure, endocrowns offer a promising restorative option (Hadeer et al. 2023).

When managing proximal carious lesions in teeth with significant structural loss, elevating the cervical margin and placing a direct composite restoration is recommended. Proximal box elevation facilitates the impression and cementation procedures by improving visibility and accessibility of the cervical margin (Soh et al. 2003). An added benefit of the PBE technique is the possibility of performing immediate dentin sealing simultaneously. This combined approach enhances bonding strength, reduces marginal leakage, and improves retention (Mertsöz et al. 2023). Research on the periodontal impact of DME demonstrates that it is well tolerated both clinically and histologically (Bertoldi et al. 2019). According to Mugri et al.’s long-term study, teeth treated with DME showed higher survival rates compared to those subjected to surgical crown lengthening (Mugri et al. 2021).

This study aimed to determine the effect of the restorative technique known as DME on the fracture strength of translucent multi-layered zirconia endocrowns used in endodontically treated premolars. The investigation was conducted in vitro to standardize procedures across all samples and allow a more detailed evaluation of the relevant variable. This approach may yield valuable insights for improving restorative protocols. In this study, human teeth were used instead of alternative models to more accurately simulate clinical conditions, including thermal conductivity, bonding behavior, modulus of elasticity, and strength (Alqahtani et al. 2023). To prevent dehydration and degradation, specimens were stored in 1% thymol solution. A bioceramic sealer compatible with resin cement and without adverse effects on polymerization or bond strength was used for obturation (Bassyouni et al. 2024).

Specimens were fixed to the mold using self-cured epoxy resin to simulate teeth within the alveolar bone, as its elastic modulus of 12 GPa closely approximates that of human bone, which is approximately 18 GPa. To create a standard butt-joint preparation 2 mm above the CEJ, the specimens were sectioned perpendicularly to their longitudinal axis to simulate the compromised condition of severely damaged endodontically treated teeth. This level enables the use of the pulp chamber's dentinal walls for macro-mechanical retention (Alshali and Attar 2023). To ensure uniformity, each endocrown was milled with consistent occlusal morphology and height (Nidarch 2015).

DME in this study was performed using a modified matrix technique to replicate clinical conditions. This matrix modification aimed to enhance performance, minimize invasive procedures, and reduce the need for surgical interventions or extractions (Dartora et al. 2021).

Monolithic zirconia endocrowns were selected in this study due to their superior mechanical performance and fatigue failure resistance compared to other ceramics, such as zirconia-reinforced lithium silicate, leucite-based ceramics, and lithium disilicate glass ceramics, regardless of the number of remaining axial walls (Demachkia et al. 2023). Sandblasting with 50 μm aluminum oxide particles and the use of acidic adhesive monomer (MDP) in both Z-Prime Plus and All-Bond Universal Adhesive enhanced bonding to zirconia by forming a chemical bond with zirconia-based ceramics (Magne 2023; Koko et al. 2022).

Thermocycling in a water bath was performed after cementation to simulate temperature and humidity variations, thereby mimicking aging in the oral environment. The number of 500 cycles was selected to provide a basic assessment of material performance under thermal stress and to maintain consistency with comparable studies following ISO protocols. However, it is important to note that 500 cycles represent a relatively short duration of clinical service, approximately equivalent to less than one month of intraoral function. Marginal adaptation was evaluated using a stereomicroscope, a non-destructive method that provides essential data on marginal quality (Soliman et al. 2022). The fracture resistance test was employed to assess the effects of restorative materials, preparation design, and bonding techniques on the durability and tensile strength of the restoration (Mahgoub et al. 2024).

For CAD/CAM ceramic restorations, marginal quality is critical, as it reduces the risk of cement degradation and secondary caries. A marginal discrepancy of up to 160 μm is considered clinically acceptable (Yildiz et al. 2013). In the present study, Groups 1 and 2 recorded marginal gap measurements of 38.39 μm and 33.04 μm, respectively. These values fall within the clinically acceptable range and are consistent with previous studies (Homaei et al. 2016a). This may be attributed to the use of CAD/CAM technology, which provides uniform and precisely fitting surfaces, thereby improving marginal adaptation. This approach reduces marginal gaps and facilitates more accurate seating of restorations (Homaei et al. 2016b; Al Mansour et al. 2015). However, this contrasts with findings by Mously et al. (2014). who reported inferior marginal quality for CAD/CAM ceramics compared to conventional methods. Similar conflicting results were observed by Azar et al. (2018). Recent comparative studies have shown that both conventional and CAD/CAM restorations exhibit comparable performance in terms of marginal adaptation (Al Hamad et al. 2019; Dolev et al. 2019).

The higher mean value in Group I (38. 39) compared to Group II (33.04) may be attributed to thermal stresses at the adhesive interface resulting from differences in the coefficients of thermal expansion among dentin, enamel, and adhesive cements (11 × 10^–6^/°C, 17 × 10^–6^/°C, and 20 × 10^–6^/°C to 80 × 10^–6^/°C, respectively). This issue is exacerbated when a composite restoration is present, as it may have a high coefficient of thermal expansion (Dawood and Al-Zordk 2021).

It is widely recognized that DME has beneficial effects (Roggendorf et al. 2012; Marchesi et al. 2014). As demonstrated by Zaruba et al. (2013), who examined the marginal integrity of ceramic inlays with subgingival boxes restored with composite resin for margin elevation, the marginal quality was comparable to that of ceramic inlays placed directly into dentin without subgingival boxes.

In contrast, Kuper et al. (2012) found that elevating the proximal box with composite increased microleakage and recurrent caries due to exposure of the hybrid layer, which led to hydrolysis of unprotected collagen. This degradation adversely affected the adhesive interface, ultimately compromising marginal integrity.

Both Groups 1 and 2 exhibited fracture resistance values within clinically acceptable limits, likely due to the favorable inherent mechanical properties of the tested ceramic material. The flexural strength of translucent multi-layered Katana zirconia blocks exceeds the maximum biting force for a single molar (725 N) (Ghajghouj and Tasar-Faruk 2019; Al-Akhali et al. 2017).

Furthermore, the design of the endocrown preparation converts occlusal forces into compressive loads across the butt joint, providing a stable surface aligned with the occlusal plane and enhancing resistance to compressive stresses. The proximal box features narrow axial walls to counteract the shear stress distributed across the box, thereby contributing to its high fracture resistance (Adel et al. 2019).

This finding is consistent with a previous study that reported no significant difference in fracture strength between groups restored with and without DME, regardless of the overlay material used (Bresser et al. 2020). Moreover, prior studies have shown that the PBE technique significantly enhances the restoration's resistance to fracture under compressive force (Dartora et al. 2018; Pedrollo Lise et al. 2017). The high fracture resistance is primarily attributed to improvements in structural support (Firouzmandi et al. 2016).

The mode of failure results (Group I: 100% catastrophic failure below the CEJ; Group II: 55.6% catastrophic failure below the CEJ, 22.2% repairable failure above the CEJ, and 22.2% repairable debonding) align with findings from Roggendorf et al. (2012), who investigated the effect of deep margin elevation in an in vitro study using indirect composite inlays. This can be explained by the use of resin for proximal box elevation, which absorbs part of the loading stress and acts as a stress breaker (Zaruba et al. 2013). Composite resin can reduce the stress on the remaining tooth structure due to its mechanical properties, which are comparable to those of human dentin (Llgenstein et al. 2015).

To promote favorable stress distribution, mesial marginal elevation using adhesive materials provides a supportive foundation beneath the margin of the ceramic endocrown, thereby mimicking the function of intact tooth tissue. This is supported by numerous studies showing that teeth restored with composite exhibit fracture resistance comparable to that of intact, unrestored teeth (Shafiei et al. 2011; Mondelli et al. 2009). Furthermore, research by (Zamboni et al. 2014) demonstrated that DME may reinforce cusps and reduce cuspal deflection, both of which contribute to increased fracture resistance. According to the present study, the differences in strength are not attributed to the DME itself but instead appear to result from the greater amount of tooth structure removed in Group II.

## Conclusion

DME has no significant effect on either marginal adaptation or fracture resistance of premolar teeth restored with multi-layered translucent zirconia endocrowns. However, DME slightly improves the failure mode.

### Limitation

A limitation of this study is the use of only 500 thermocycles, which does not fully replicate the long-term thermal stresses and material degradation encountered intraorally. Future studies employing a higher number of thermocycles and additional aging protocols are needed to better simulate intraoral conditions.

## Data Availability

All data generated or analyzed during this study are included in this published article.

## Declarations

### Clinical trial number

Not applicable (in vitro study).
